# Regulation of CEACAM Family Members by IBD-Associated Triggers in Intestinal Epithelial Cells, Their Correlation to Inflammation and Relevance to IBD Pathogenesis

**DOI:** 10.3389/fimmu.2021.655960

**Published:** 2021-07-29

**Authors:** Gonzalo Saiz-Gonzalo, Naomi Hanrahan, Valerio Rossini, Raminder Singh, Mary Ahern, Maebh Kelleher, Shane Hill, Ruairi O’Sullivan, Aine Fanning, Patrick T. Walsh, Seamus Hussey, Fergus Shanahan, Ken Nally, Caitriona M. O’Driscoll, Silvia Melgar

**Affiliations:** ^1^APC Microbiome Ireland, University College Cork, National University of Ireland, Cork, Ireland; ^2^Department of Medicine, University College Cork, National University of Ireland, Cork, Ireland; ^3^School of Biochemistry and Cell Biology, University College Cork, National University of Ireland, Cork, Ireland; ^4^School of Pharmacy, University College Cork, National University of Ireland, Cork, Ireland; ^5^Department of Clinical Medicine, School of Medicine, Trinity College Dublin, Dublin, Ireland; ^6^National Children’s Research Centre, Our Lady’s Children’s Hospital, Dublin, Ireland; ^7^Department of Pediatric Medicine, RCSI University of Medicine and Health Sciences, Dublin, Ireland

**Keywords:** epithelial cells, carcinoembryogenic antigen cellular adhesion molecules, polysorbate-80, short-chain fatty acids, adherent-invasive *Escherichia coli*, pro-inflammatory cytokines, inflammatory bowel diseases, Tofacitinib

## Abstract

Carcinoembryogenic antigen cellular adhesion molecules (CEACAMs) are intercellular adhesion molecules highly expressed in intestinal epithelial cells. CEACAM1, -3, -5, -6, -7 are altered in patients suffering from colon cancer and inflammatory bowel diseases (IBD), but their role in the onset and pathogenesis of IBD is not well known. Herein, we aim to correlate CEACAM1, -3, -5, -6, -7 expression to the degree of inflammation in pediatric and adult IBD colon biopsies and to examine the regulation of CEACAMs on human intestinal epithelial cell lines (C2BBe1/HT29) by different IBD-associated triggers (cytokines, bacteria/metabolites, emulsifiers) and IBD-drugs (6-Mercaptopurine, Prednisolone, Tofacitinib). Biopsies from patients with pediatric Crohn’s disease (CD) and adult ulcerative colitis (UC, active/inactive disease) showed a significant increase in CEACAM3, -5, -6 expression, while CEACAM5 expression was reduced in adult CD patients (active/inactive disease). Intestinal epithelial cells cultured with a pro-inflammatory cytokine cocktail and Adherent-invasive *Escherichia coli* (AIEC) showed a rapid induction of CEACAM1, -5, -7 followed by a reduced RNA and protein expression overtime and a constant expression of CEACAM3, correlating with IL-8 expression. Cells cultured with the emulsifier polysorbate-80 resulted in a significant induction of CEACAM3, -5, -6, -7 at a late time point, while SCFA treatment reduced CEACAM1, -5, -7 expression. No major alterations in expression of CEACAMs were noted on cells cultured with the commensal *Escherichia coli* K12 or the pathogen *Salmonella typhimurium*. IBD drugs, particularly Tofacitinib, significantly reduced cytokine-induced CEACAM1, -3, -5, -6, -7 expression associated with a reduced IL-8 secretion. In conclusion, we provide new evidence on the regulation of CEACAMs by different IBD-associated triggers, identifying a role of CEACAMs in IBD pathogenesis.

## Introduction

Inflammatory Bowel Disease (IBD), encompassing Ulcerative Colitis (UC) and Crohn’s Disease (CD), is a chronic inflammatory condition affecting the gastrointestinal tract with unknown etiology and accelerating incidence in the western world in both children and adults (0.5–24.5 per 100,000 person/year) (1). In children, the overall disease course of CD is often more severe, especially in those who are diagnosed before the age of 10 years, which can affect their growth, psychological well-being, nutrition, and schooling (2). Long-term chronic inflammation is a risk factor for the development of colon cancer, especially in patients with UC (3, 4). There is currently no cure for IBD, and available therapies treat the symptoms of the disease and maintain patients in remission. However, efficacy of treatment is varied, and up to 30% of the patients become non-responders to any treatment (5). Among anti-inflammatory drugs used in IBD patients are methylprednisolone, a potent steroid, and Tofacitinib, a Janus kinase (JAK)-inhibitor (6, 7), while drugs used for remission maintenance include the immunosuppressants azathioprine and 6-mercaptopurine (6-MP) (8).

Although the etiology of IBD is unknown, the collective evidence indicates that IBD is widely impacted by genetic, inflammatory, and environmental factors and gut microbiota (9, 10). Many factors have been identified as likely contributors to the onset and severity of the disease. These include a high-fat diet and dietary emulsifiers widely affecting the microbiota by promoting a pro-inflammatory bacterial environment, as shown in experimental and in *ex vivo* human fecal models (5, 11). A reduced microbiota diversity has been associated with IBD, and some bacteria strains including Adherent-invasive *Escherichia coli* (AIEC) and *Salmonella typhimurium* have been identified in these patients (12–14). For example, up to 40% of patients with CD harbor the pathobiont AIEC in their mucosa, and several strains including LF82 and HM605 have been isolated and characterized from the ileal (LF82) and colonic (HM605) mucosa of patients with CD, indicating a role of AIEC in CD pathogenesis (13, 14).

Carcinoembryogenic antigen cellular adhesion molecules (CEACAMs) are a group of intercellular adhesion molecules that act as modulators of key cellular processes, including cell adhesion, cell differentiation, cell proliferation, and cell survival (15). CEACAMs are widely expressed in the body, including the gut, and found on both epithelial and immune cells (16, 17). CEACAMs found in mucosal tissues include CEACAM1, -5, -6, -7 all found in the gut (17, 18) and CEACAM3 mostly found in the lung (19). In addition, CEACAM20 was recently reported to be localized in epithelial cells in the murine small intestine, expressed in epithelial cell lines, and its expression regulated by commensal bacteria (20). However, its role in human colon or IBD is currently unknown. CEACAM5 and -7 display selective epithelial expression, while CEACAM1, -3, -6, -20 display broad expression in the body and in different cell types (18). CEACAM3 is expressed mostly in immune cells in the lung and acts as a receptor for certain bacteria including *Neisseria gonorrhoeae (* 18, 21). Clinically, CEACAM5 is widely used as a marker of tumor progression in colonic malignancies, and an elevation in CEACAM5 levels is generally seen as a sign of a large tumor burden and poor prognosis (22). Alterations in CEACAM1, -5, and -6 have been reported in IBD and in epithelial cells exposed to IFNγ (20). A reduction in CEACAM1 and -5 and an induction of CEACAM6 have been reported in CD (3, 23), while an increase in CEACAM5 expression has been reported in UC (24). Neither CEACAM7 nor CEACAM3 has previously been studied in IBD, although a reduction in CEACAM7 and CEACAM3 was reported in patients in stage II recurrent and non-recurrent colorectal cancer (25) and in the post-operative phase of patients with colorectal cancer, respectively (26). As several of these CEACAMs are localized on the apical surface of the epithelial cells, they can provide an accessible handle for incoming bacteria. Indeed, CEACAM1, -3, -5, and -6 have been shown to act as receptors for specific bacteria and bacterial ligands (17). Interestingly, CEACAM6 has been identified as a receptor for the CD-pathobiont AIEC, but its expression in naïve IBD patients or regulation by commensal or pathogenic bacteria has not been studied yet. Overall, there is an indication that CEACAMs might be enrolled in the initiation and progression of inflammation in IBD, specially by regulating the innate immune and epithelial responses in the intestine. Based on the collected literature on CEACAMs and IBD, the aims of this study are to (1) investigate the expression of CEACAMs in colonic biopsies of patients with pediatric and adult (active and inactive disease) UC and CD and their correlation to inflammation, and (2) to investigate how certain IBD-associated stimuli and IBD-therapeutic treatments regulate the expression of CEACAMs on intestinal epithelial cells (IECs).

## Materials and Methods

### Bacterial Strains and Growth

The commensal *E. coli* K12, the CD-pathobiont AIEC-HM605, and the pathogen *S. typhimurium* were grown overnight in Lysogeny Broth (LB) (LENNOX, Sigma-Aldrich), with the LB-medium supplemented with 100 μg/ml Ampicillin (Thermo Fisher Scientific) for AIEC. For co-culture experiments, an estimate of the number of alive bacteria was performed to calculate the multiplicity of infection (MOI) 10:1 for the bacteria to C2BBe1 cells. For collection of conditioned media (CM), *E. coli* K12, AIEC-HM605, and *S. typhimurium* were grown to log phase, following which bacteria broth was collected, centrifuged, filter sterilized (0.45 μm), pH adjusted (7–7.5), frozen, and stored at −80°C.

### Human Intestinal Epithelial Cell Lines

C2BBe1 and HT29 human intestinal epithelial cell lines were obtained from American Type Culture Collection (Rockville). HT29 cells were grown in McCoy’s 5A Medium Modified, with L-glutamine and sodium bicarbonate (Sigma-Aldrich). C2BBe1 cells were grown in DMEM (Dulbecco’s Modified Eagle’s Medium), high glucose (Sigma-Aldrich). Both media were supplemented with 10% of Fetal Bovine Serum (FBS) and 1% of penicillin/streptomycin (Pen/Strep, Sigma-Aldrich). DMEM media was additionally supplemented with 0.01 mg/ml human Apo-transferrin (Sigma-Aldrich). Both cell lines were passaged once they reached 70–80% confluency and grown at 37°C, 5% CO_2_.

### Co-Culture of Human Intestinal Epithelial Cells With the Dietary Emulsifier Polysorbate 80

HT29 cells were seeded in 24-well plates at a density of 1 × 10^5^ cells/well (for qRT-PCR analysis) and in 96-well plates at 20,000 cells per well (for viability/proliferation assays) in McCoy’s 5A Medium Modified culture medium and co-cultured with polysorbate 80 (p80) at a concentration of 0.25% w/v diluted in the same media. HT29 cells were cultured in 0.25% p80 for 1, 2, 4, and 6 hours (h) and cells collected at each time point, washed with phosphate-buffered saline (PBS, Sigma), and lysed, with lysates frozen at −80°C for PCR analyses. All conditions were performed in triplicates. For RNA extraction, the triplicates of each condition were extracted and pooled for gene expression analysis. At the 6 h time point, HT29 cells treated with 0.25% p80 were assayed for cell proliferation (BrdU Cell Proliferation Assay, Cell Signaling Technology, Ireland) and cell viability (CellTiter-Blue, Viability Assay, Promega Corporations, MyBio, Ireland) following the manufacturer’s instructions.

### Co-Culture of Human Intestinal Epithelial Cells With Whole Bacteria or Pro-inflammatory Cytokine Cocktail

C2BBe1 cells were seeded in 24-well plates at a density of 1 × 10^5^ cells/well in full DMEM culture medium. Once cells reached confluency, they were cultured in serum and antibiotic-free DMEM overnight (12–16 h) followed by co-culture for 3 h with the commensal *E. coli* K12, the CD-pathobiont AIEC, the pathogen *S. typhimurium* [all bacteria strains added at MOI 10:1] as recently described (27), or a pro-inflammatory cytokine cocktail [consisting of recombinant human (rh) TNF-α (20 ng/ml), rh IFN-γ (10 ng/ml), rh IL-1β (10 ng/ml), all purchased from Peprotech], followed by 3× wash in PBS + 1% Pen/Strep, and a further incubation in full culture DMEM media for 2 (T2), 5 (T5), and 13 (T13) h post infection/cytokine treatment. At each time point, cells were washed with PBS and lysates stored at −80°C for RT-qPCR analyses. All conditions were performed in triplicates.

### Co-Culture of Human Intestinal Epithelial Cells With Conditioned Bacteria Media or Short-Chain Fatty Acid (SCFA) Mixture

C2BBe1 cells were seeded in 24-well plates at a density of 1 × 10^5^ cells/well in full DMEM culture medium, followed by overnight culture (12–16 h) in serum-free media and co-cultured for 24 h at a 1:1 ratio with CM collected from the commensal *E. coli* K12, the pathobiont AIEC, and the pathogen *S. typhimurium* or with a SCFA mixture containing 5 mM acetate, 2 mM propionate, and 5 mM butyrate. At the end of the experiment, lysates of the cells were collected and stored at −80°C for PCR analyses. All treatments were performed in triplicates.

### Treatment of Human Intestinal Epithelial Cells With Pro-inflammatory Cytokine Cocktail and IBD Drugs

C2BBe1 cells were seeded in 24-well plates at a density of 1 × 10^5^ cells/well in full culture medium, followed by overnight culture (12–16 h) in serum-free media. To investigate the impact of IBD-drug treatment on cytokine-induced CEACAM expression, C2BBe1 cells were pretreated for 1 h with the IBD drugs—Tofacitinib 10 mM (Tebu-bio Ltd, UK), Methylprednisolone 10 mM (Sigma), Mercaptopurine (6-MP) 5 mM (Sigma)—followed by addition of the cytokine cocktail [rhTNF-α (20 ng/ml), rh IFN-γ (10 ng/ml), rh IL-1β (10 ng/ml)] for 3 h, subsequently washed in PBS and further incubated in full media containing each corresponding drug treatment for 2 (T2) and 13 (T13) h. All drugs were diluted in DMSO, and control wells received 0.1% DMSO. All treatments were performed in triplicates. At T2 and T13, cells were washed with PBS, and lysates of the cells and supernatant were collected and stored at −80°C for further PCR and ELISA analysis, respectively.

### Real-Time qRT-PCR Gene Expression Analysis

Total RNA was isolated from biopsy samples and cells using a RNeasy Mini Kit (QIAGEN, Hilden, Germany), and DNA digestion was performed using the Turbo DNA-free Kit (Thermo Fischer Scientific, Ireland), following the manufacturers’ instructions. For RNA extraction from the cell line experiments, the triplicates of each condition were extracted and pooled for gene expression analysis. cDNA was synthesized using 1 μg total RNA using Reverse Transcriptase enzyme provided by Transcription First Strand cDNA Synthesis Kit (Roche, Ireland). PCR primers (Eurofins, Ireland) and probes (Roche) were designed using the Universal ProbeLibrary Assay Design Center (https://www.roche-applied-science.com/sis/rtpcr/upl/adc.jsp; Roche Applied Science, Indianapolis, IN, USA). PCR reactions were performed in duplicates/triplicates by using the LightCycler 480 system (Roche Applied Science). Positive and negative controls were also included. The genes analyzed included CEACAM1, CEACAM3, CEACAM5, CEACAM6, CEACAM7, CEACAM20, CCL20, and IL-8/CXCL8 (see **Supplementary Table 3** for primer and probe sequences). β-actin was used as housekeeping gene. The 2^−ΔΔ^ Ct method was used to calculate the relative changes in each of the genes relative to the expression of β-actin determined from real-time qRT-PCR experiments (28, 29).

### ELISA

The cytokine levels were measured using the Human CCL20/MIP-3α (DY360) and IL-8/CXCL8 (DY208) Duo Set ELISA kits (R&D systems) in supernatants collected from the above experiments, according to the manufacturer’s instructions.

### Flow Cytometry

C2BBe1 cells were seeded in six-well plates at a concentration of 500,000 cells/well in full DMEM, followed by treatment with the pro-inflammatory cocktail [(“rhTNF-α IFN-γ rh IL-1β”) as outlined in the section *Co-culture of Human Intestinal Epithelial Cells With Whole Bacteria or Pro-inflammatory Cytokine Cocktail*] for 3 h and further incubated in full culture DMEM media for 5 (T5) and 8 (T13) h post-treatment. Cells were then washed with sterile 1 × PBS and incubated with 1 ml of trypsin for 10 min at 37°C. Cells were collected in 1 ml of completed DMEM media and centrifugated 5 min at 200 × g. C2BBe1 cells were washed once in PBS supplemented with 1% bovine serum albumin (BSA) and 0.1% sodium azide. Non-specific binding of antibodies (Abs) to Fc receptors was blocked by pre-incubation of cells with BD Pharmingen™ Human BD Fc Block™ (cat nr. 564220) in monoclonal Abs (0.5 ng mAb per 10^6^ cells). The cells were then incubated with 2.5 µg of the primary Abs (Human CEACAM1/CD66a, Mouse IgG2B, Clone #283340; Human CEACAM3/CD66d, Sheep IgG; Human CEACAM5/CD66e, Mouse IgG2A, Clone #487609, all from R&Systems, Biotechne) for 20 min at 4°C and washed twice. Cells were subsequently incubated with a relevant secondary mAb (FITC anti-mouse IgG2b, cat nr 406705; APC anti-mouse IgG2a, cat nr 407109, both from Biolegend; Sheep IgG PE-conjugated, cat nr F0126, R&D Systems, Biotechne) for 20 min at 4°C and washed twice. A final live/dead staining step was performed using the fixable viability dye eF780 conjugated (eBioscience cat. 65-0865-14) following the manufacturer’s instructions. Multicolor flow cytometry analyses were performed using the three laser (405, 488, and 460nm) BD Celesta FACS Analyser. Data were analyzed using FCS Express V5 DeNovo software (Pasadena, CA, USA).

### Western Blotting

Western blotting was performed according to Woznicki et al. (29), with some modifications. Briefly, C2BBe1 cells were seeded in six-well plates at a density of 500,000 cells/well and treated for 3 h with the pro-inflammatory cocktail (rhTNF-α/IFN-γ/IL-1β as outlined in the section *Co-culture of Human Intestinal Epithelial Cells With Whole Bacteria or Pro-inflammatory Cytokine Cocktail*) for 3 h (T0), followed by further incubation in full culture DMEM media for 2 (T2) and 5 (T5) h post-treatment. Cells from three wells per condition were pooled, washed in cold PBS, and lysed on ice for 20 min in RIPA lysis buffer [50 nM Tris, pH 8.0, 150 nM NaCl, 1% NP40, 0.5% sodium deoxycholate, and 0.1% sodium dodecyl sulphate (SDS)] supplemented with Pierce Halt Protease Inhibitor Cocktail, sodium orthovanadate, sodium pyrophosphate, and glycerophosphate (all 1:1,000, from Thermo Fisher Scientific). After centrifugation (20,817 × g) for 15 min at 4°C, the protein concentration of cell lysates was determined using a BCA assay, as per manufacturers’ guidelines (Thermo Fisher Scientific). The protein preparation (80 μg) was denaturated at 75°C for 10 min in 4× LSD sample Loading Dye buffer (Invitrogen), 10× Reducing agent buffer (Invitrogen). Samples were run on Bolt 4–12% gradient Bis-Tris Plus Gels (cat nr. NW04122Box, Thermo Fisher Scientific) at 120 V for 1 h and transferred to a Immobilon PVDF membrane (Millipore) for 1 h. Membranes were washed in Tris Buffer Saline (TBS) and blocked for 1 h in 5% skimmed milk (0.5% Tween20 in TBS), followed by incubation overnight and rocking at 4°C with the primary antibodies [human CEACAM6/CD66c (Mouse IgG2A, Clone #439424, cat nr. MAB3934-SP, R&D-Biotechne); human CEACAM7 (Sheep IgG, cat nr. AF4478, R&D-Biotechne); β-actin (Mouse IgG2b, cat nr. 8H10D10, Cell Signaling Technology], diluted at 1:1,000 in 5% BSA/TBS-T. The membranes were washed and incubated with the corresponding secondary antibody [anti-Sheep IgG HRP-conjugated, cat nr. HAF016, R&D-Biotechne; anti-Mouse IgG HRP-conjugated, Cell Signaling Technology (7076)] at 1:1,000 dilution in 5% BSA/TBS-T, for 1 h at room temperature. Western bright quantum was used for detection, and LAS 3000 Imager and software was used to acquire images. For re-probing, samples were stripped using stripping buffer, washed, blocked with 5% skimmed milk/TBS-T as above, and probing repeated.

### Statistical Analysis

The data collected are presented as a mean ± standard error of the mean (SEM) unless otherwise stated. The data are analyzed by an unpaired t-test to compare the difference between two groups and by ANOVA with *post-hoc* analysis to compare the difference between more than two groups, and Spearman was used for correlation analysis. A p-value of <0.05 was considered significant, and all analyses were carried out using the computer software program Prism (GraphPad Software, San Diego, CA, USA).

### Ethical Considerations: Adult and Pediatric Patients and Tissue Samples

Human adult and pediatric samples from both sexes were used in this study. All adult patients were recruited from the gastroenterology clinics at Cork University Hospital, Cork, Ireland. All IBD patients and control subjects gave written informed consent. The study protocol, including all procedures, was approved by the University College Cork Clinical Research Ethics Committee of Cork Teaching Hospitals (APC015). The adult IBD patients were characterized as having an active and inactive disease at the time of endoscopy. Active disease was defined as the presence of symptoms and inflammatory changes evident on endoscopy and confirmed by histology. The severity was not scored (because the research question did not call for correlation with severity other than a comparison of active *v* inactive [(remission) - inflammation *versus* non-inflamed)]. Inactive patients were those in remission and defined as being completely asymptomatic and with no evidence for abnormality on endoscopy, which was being performed for surveillance purposes, not for assessment of disease activity. All pediatric patients and control participants were recruited with consent under the Determinants and Outcomes of Children and Adolescents with IBD (DOCHAS) study at the gastroenterology unit at Our Lady’s Children’s Hospital Crumlin, under approval from the institutional Research Ethics Committee (GEN/193/11). All patients have been diagnosed according to international pediatric standards (Porto criteria), following which, each case was rigorously phenotyped using the pediatric-specific Paris classification of IBD. Established pediatric evidence-based indices are used to document clinical disease activity—a Physician Global Assessment score for each case; the Pediatric Crohn’s Disease Activity Index (PCDAI) for CD and Pediatric Ulcerative Colitis Activity Index (PUCAI) for UC. All biopsies were obtained from patients with informed consent as part of routine diagnostic evaluation. The experiments were performed in accordance with the World Medical Association Declaration of Helsinki Ethical Principles for Medical Research.

Detailed clinical information on the pediatric and adult patients included in this study are depicted in **Supplementary Tables 1**, **2**, respectively. A total of 12 pediatric and 19 adult colonic biopsy samples were obtained from patients diagnosed with IBD. Six pediatric and 14 adult samples of healthy colonic tissue were used as controls. The pediatric group was divided in three groups: healthy controls (n=6, 5 males, 1 female); biopsies from patients with pediatric Ulcerative colitis (n=9; 6 males, 3 females); and biopsies from patients with pediatric active Crohn’s Disease (n=12; 7 males, 5 females). Pediatric patients were treatment-naive at the time of collection. The adult samples were divided into five groups: healthy biopsies (n=14; 4 males, 10 females); biopsies from patients with active UC (n=19; 10 males, 9 females); biopsies from patients with inactive UC (patients who are in remission) (n=19; 10 males, 9 females); biopsies from patients with active CD (n=12; 3 males, 9 females); and biopsies from patients with inactive CD (n=10; 3 males, 7 females). Patients undergoing treatment are represented as aminosalicylates, immunosuppressant, steroids, anti-TNFs, none, and other (see details in **Supplementary Table 2**). Information on treatment and length of disease duration was not available for four patients (4 of 19 patients, 21%) in the UC active group and for two patients (2 of 12 patients, 16.7%) in the CD active group.

## Results

### Increased Expression of CEACAM3, CEACAM5, and CEACAM6 in Colonic Biopsies of Pediatric Patients With Crohn’s Disease

**Figure 1:**
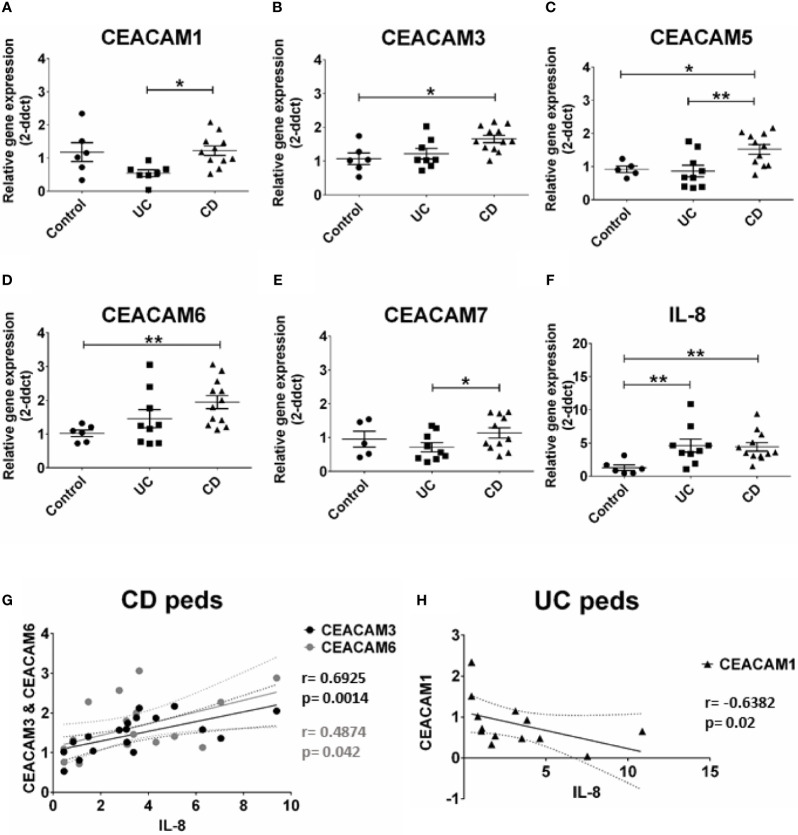
CEACAMs gene expression in pediatric colonic mucosa of patients with Ulcerative colitis (UC) and Crohn’s disease (CD) and controls. Gene expression of **(A)** CEACAM1, **(B)** CEACAM3, **(C)** CEACAM5, **(D)** CEACAM6, **(E)** CEACAM7, **(F)** IL-8 as determined by RT-qPCR. **(G)** Spearman correlation on relative mRNA levels between IL-8 and CEACAM3 and CEACAM6 in pediatric colonic CD samples and of **(H)** IL-8 and CEACAM1 in pediatric colonic UC. n=9, Ulcerative Colitis (UC); n=12 Crohn’s Disease (CD); and n=6 controls. An ANOVA test with *post-hoc* analysis was used to analyze gene expression data and Spearman correlation test. *P < 0.05, **P < 0.01.

Previous studies have identified an altered expression in CEACAMs in biopsies from patients with IBD (9). In this study we expanded these findings by examining whether colonic CEACAMs are altered in children with IBD without any previous treatment and whether changes in CEACAM expression correlate to the degree of inflammation. The gene expression of CEACAM3, -5, -6 is significantly increased in colon biopsies of pediatric patients with CD, while no alterations are detected in pediatric UC biopsies, when compared to controls (**Figures 1B–D**). When pediatric CD are compared to pediatric UC samples, a significant increase in CEACAM1, -5, -7 expression is observed in pediatric biopsies of patients with CD (**Figures 1A, C, E**). Contrary to findings in the murine intestine (20), CEACAM20 is not detected in any of the pediatric colonic samples assayed (data not shown). When the IBD pediatric biopsies are analyzed based on clinical disease activity, no major difference between UC *versus* CD is detected on the expression of the CEACAMs investigated (data not shown). When we correlate the expression of CEACAMs to the pro-inflammatory cytokine IL-8 (**Figure 1F**), a positive correlation is observed for CEACAM3, -6 in pediatric CD samples (r = 0.6925, p = 0.0014 for CEACAM3 and r = 0.4874; p = 0.042 for CEACAM6, respectively, **Figure 1G**) and a negative correlation for CEACAM1 (r = -0.6382, p = 0.02) in the pediatric UC samples (**Figure 1H**).

### Increased Expression of CEACAM3, CEACAM5, and CEACAM6 in Colonic Biopsies of Adult Patients With Ulcerative Colitis

**Figure 2:**
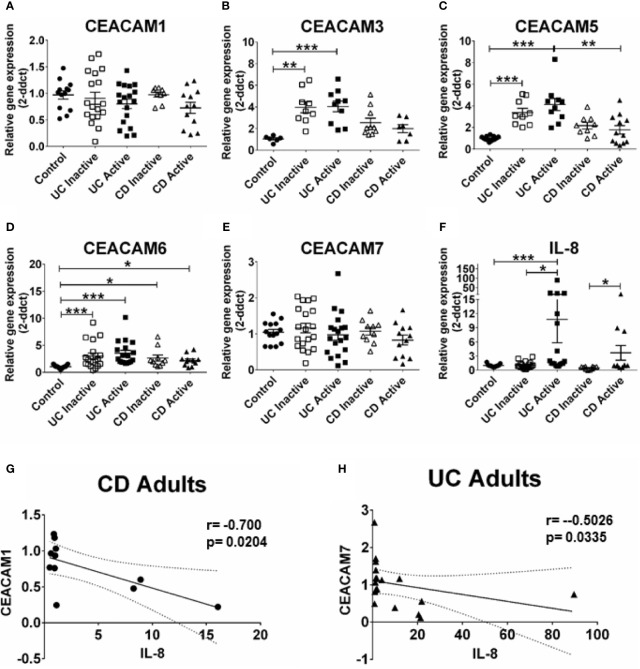
CEACAMs gene expression in adult colonic mucosa of patients with Ulcerative colitis (UC) and Crohn’s disease (CD) with active and inactive disease and controls. Gene expression of **(A)** CEACAM1, **(B)** CEACAM3, **(C)** CEACAM5, **(D)** CEACAM6, **(E)** CEACAM7, **(F)** IL-8 as determined by RT q-PCR. **(G)** Spearman correlation on relative mRNA levels between IL-8 and CEACAM1 in adult CD Active samples and of **(H)** IL-8 and CEACAM7 in adult colonic UC. n=10–18, active Ulcerative Colitis (UC); n=10–19, inactive UC; n=7–11 active Crohn’s Disease (CD); n=10 inactive Crohn’s Disease and n=6–14 controls. An ANOVA test with *post-hoc* analysis was used to analyze gene expression data and Spearman correlation test. *P < 0.05, **P < 0.01, ***P < 0.001.

Next, we ask whether the alterations in CEACAM gene expression observed in pediatric samples are present in adult IBD samples and whether these alterations are associated to the degree of inflammation in the tissue (measured by IL-8 expression). For this purpose, we examined the expression of the same CEACAM genes in colon biopsies from adult patients with active and inactive UC and CD. Our data show a significant expression of CEACAM3, -5, -6 in colon biopsies from adult patients with UC, regardless of disease state, when compared to controls (**Figures 2B–D**). Consistent with previous reports (8), an increased expression in CEACAM6 is detected in adult CD samples, regardless of disease state, and in adult UC samples, with both active and inactive disease, when compared to controls (**Figure 2D**). Similar to findings in the pediatric biopsies, no change in the expression of CEACAM1, -7 is observed in CD or UC samples, regardless of disease status, when compared to controls (**Figures 2A, E**). In agreement with the findings in the pediatric samples, CEACAM20 is not detected in any of the adult groups analyzed (data not shown). When CD samples are compared to UC samples, a significant reduction in CEACAM5 expression is detected in samples from CD active when compared to UC active (**Figure 2C**). When we correlate the expression of CEACAMs to IL-8 (**Figure 2F**) in the adult biopsies with active disease, a negative correlation is observed for CEACAM1 in adult CD samples (r = −0.700, p = 0.0204, **Figure 2G**) and for CEACAM7 (r = −0.5026; p = 0.0335) in the adult UC samples (**Figure 2H**).

### The Dietary Emulsifier p80 Induces Cell Death and the Expression of CEACAM3, CEACAM5, CEACAM6, and CEACAM7 Over Time in Intestinal Epithelial Cells

**Figure 3:**
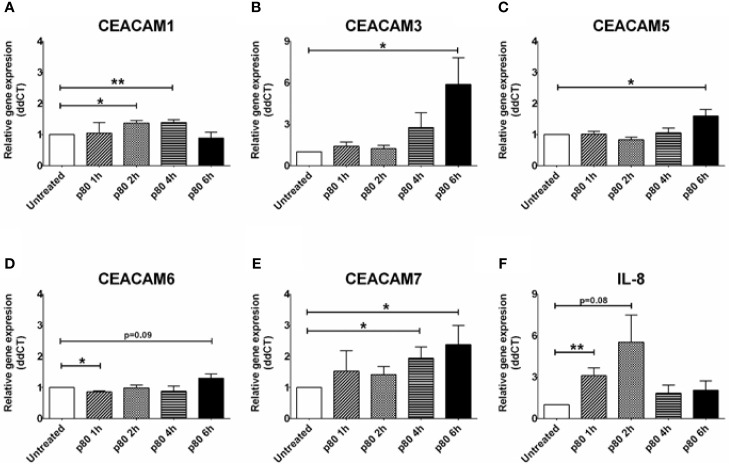
Relative gene expression profile in HT29 cells treated with 0.25% polysorbate 80 (p80) for 1, 2, 4, and 6 h for analysis on gene expression of **(A)** CEACAM1, **(B)** CEACAM3, **(C)** CEACAM5, **(D)** CEACAM6, **(E)** CEACAM7, **(F)** IL-8 as determined by RT-qPCR. Graphs are representative of two to four independent experiments with pooled triplicate samples and qRT-PCR assay performed in duplicates. β-actin was used as a housekeeping gene, and results are expressed as fold change. A Student t-test was used to analyze gene expression data. *P < 0.05, **P < 0.01 when compared to untreated cells.

Next, we explore whether environmental risk factors associated with IBD such as dietary components might affect the expression of CEACAMs in IECs. Recent studies have shown that the dietary emulsifier p80 has a negative effect on the microbiota resulting in development of metabolic syndrome, colitis, and colon cancer in experimental models (5, 11). The direct effect of dietary emulsifiers on IECs is yet to be studied. Therefore, we optimized an *in vitro* system to measure the effect of p80 on HT29 cells over time. A concentration higher than 0.5% p80 is almost 100% toxic to the cells after 24 h culture (data not shown), while 0.25% p80 provoke approx. 27% reduction in viability and an approx. 12% reduction in proliferation in HT29 cells over a shorter co-culture time (6 h, data not shown). Using 0.25% p80 in a kinetic study in HT29 cells, we observe a significant increase in CEACAM1 at 2 and 4 h time point, with a concomitant reduction at 6 h time point (**Figure 3A**). In contrast, the expression of CEACAM3, -5, -6, -7 is slightly induced at 4 h time point and significantly elevated at 6 h time point (**Figures 3B–E**). The latter time point correlates to the reduction in cell viability observed in these cells but not to an induction in inflammation, since a slight elevation in IL-8 is only found at the early 2 h time point (**Figure 3F**).

### The CD-Pathobiont AIEC Induces the Expression of CEACAM1, CEACAM3, CEACAM5, and CEACAM7 in a Time-Dependent Manner

**Figure 4:**
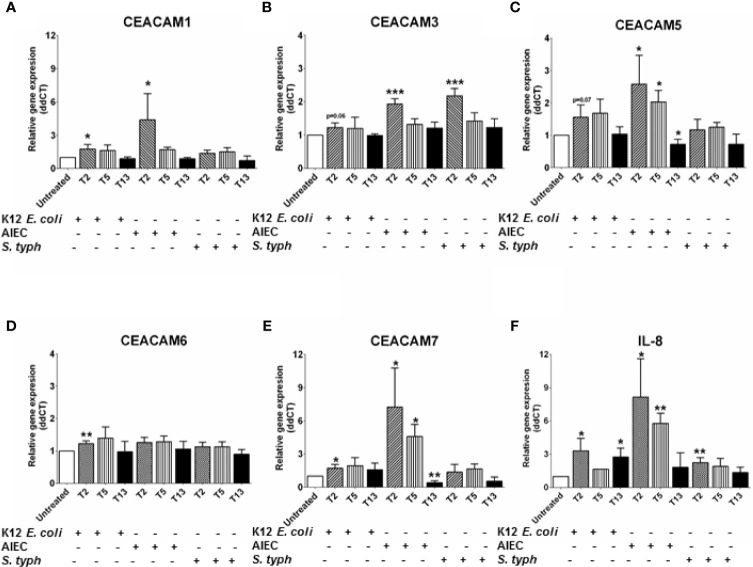
Relative gene expression profile in C2BBe1 cells co-cultured for 3 h with whole bacteria—*Escherichia coli* K12 (K12 *E. coli*), Adhesion and Invasive *E. coli* (AIEC)-HM605, *Salmonella typhimurium* (*S. typh*) at a MOI 10:1 and untreated. After the 3 h co-culture, cells are washed in medium containing antibiotics followed by further culture in full culture media for 2 (T2), 5 (T5), and 13 (T13) h. Samples are collected at each time point for analysis on gene expression of **(A)** CEACAM1, **(B)** CEACAM3, **(C)** CEACAM5, **(D)** CEACAM6, **(E)** CEACAM7, **(F)** IL-8 as determined by RT q-PCR. Graphs are representative of two to four independent experiments with pooled triplicate samples and qRT-PCR assay performed in duplicates. β-actin was used as a housekeeping gene, and results are expressed as fold change. A Student t-test was used to analyze gene expression data. *P < 0.05, **P < 0.01, ***P < 0.001 when compared to untreated cells.

An observed reduction in bacterial diversity and the identification of certain pathobionts including AIEC have provided evidence on the role of the microbiota in IBD pathology (27). CEACAMs can act as receptors for certain intestinal bacteria, e.g., AIEC can be recognized by CEACAM6; *S. typhimurium* can bind to CEACAM1, -5, -6 (12, 19), and CEACAM1, -20 are regulated by Gram-positive bacteria in mice (20). However, less is known about how specific bacterial (commensal, pathobionts, or pathogens) strains or bacterial metabolites regulate the expression of CEACAMs on IECs. For this purpose, we performed a kinetic study to assess the expression of CEACAM1, -3, -5, -6, -7, -20 in IECs co-cultured with whole bacteria of the commensal *E. coli* K12 strain, the pathobiont AIEC-HM605, and the pathogen *S. typhimurium* and their conditioned media (CM) and the health-promoting metabolite SCFA. IECs are co-cultured with whole bacteria for 3 h followed by analysis of CEACAM gene expression at T2, T5, and T13, while IECs are co-cultured with bacterial-derived CMs and SCFAs for 6 and 24 h followed by CEACAM assessment. AIEC-HM605 infection induces the highest expression of CEACAM1, -3, -5, and -7 2 h post-infection (T2), with a reduction in CEACAM1, -5, -7 over time (**Figures 4A–E**). A similar pattern of expression is observed for AIEC-induced IL-8 expression (**Figure 4F**). The commensal *E. coli* K12 weakly induces the expression of CEACAM1, -3, -5, -6, -7, as well as IL-8 at T2, T5, and T13 (**Figures 4A–F**); while the pathogen *S. typhimurium* significantly induces the expression of CEACAM3 and IL-8 at the T2 time point (**Figures 4B, F**). Contrary to the AIEC-induced expression of CEACAMs noted above, CM from AIEC weakly induces the expression of CEACAM1, while CM from *E. coli* K12 induces the expression of all CEACAMs assessed in this study, and CM from *S. typhimurium* induces the expression of CEACAM3, -7 (**Table 1**). Treatment of IECs with SCFAs significantly reduces the expression of CEACAM1, -5, -7 and IL-8 (**Table 1**).

**Table 1:** CEACAM expression in intestinal epithelial cells cultured with bacterial conditioned medium with bacterial conditioned medium and SCFA.

Gene of interest^a^	Stimuli^b^					
	LB broth	CM-	CM-	CM-	Control	SCFA mix
		*E. coli* K12	AIEC	*S. typhimurium*	(PBS)	
**CEACAM1**						
** 6 h**	–	8.3	1.3 ± 0.3	1.7 ± 0.1	**-**	0.4 ± 0.04*
** 24 h**	2.0 ± 0.02*	4.5 ± 0.4^#^	2.7 ± 0.2^#^	1.8 ± 0.1	1.0 ± 0.1	0.4 ± 0.08*
**CEACAM3**						
** 6 h**	–	8.0 ± 0.6^#^	3.8 ± 1.2	5.0 ± 0.1^#^	**-**	5.0 ± 1.8**
** 24 h**	2.6 ± 0.9**	4.8 ± 1.3	2.6 ± 0.6	2.4 ± 0.3	1.0 ± 0.06	1.1 ± 0.2
**CEACAM5**						
** 6 h**	–	2.4 ± 0.6	1.1 ± 0.4	2.5 ± 0.2	**-**	0.4 ± 0.1***
** 24 h**	3.4 ± 0.7**	9.9 ± 2.1	3.6 ± 0.2	2.9 ± 0.3	1.0 ± 0.02	0.8 ± 0.1 **
**CEACAM6**						
** 6 h**	–	1.3 ± 0.6	1.1 ± 0.02^#^	1.9 ± 0.03	**-**	0.8 ± 0.1
** 24 h**	4.6 ± 0.5***	7.3 ± 0.7	4.4 ± 0.7	2.9 ± 0.6	1.0 ± 0.04	1.3 ± 0.2
**CEACAM7**						
** 6 h**	**-**	–	1.8 ± 0.5	4.5 ± 0.3	**-**	0.7 ± 0.1**
** 24 h**	2.2 ± 0.5*	7.3 ± 1.05^#^	2.1 ± 0.08	2.0 ± 0.3	1.0 ± 0.01	0.6 ± 0.05***
**IL-8**						
** 6 h**	–	5.0 ± 1.6^#^	3.4 ± 1.4^#^	2.4 ± 0.4	**-**	0.6 ± 0.2*
** 24 h**	0.9 ± 0.2	3.6 ± 0.5^##^	1.9 ± 0.3^#^	1.7 ± 0.3	1.0 ± 0.1	0.6 ± 0.2*

a Gene of interest assayed after epithelial cell culture with respective stimuli for 6 and 24 h (h).

b Co-culture stimuli including LB broth, conditioned media (CM) from Escherichia coli (E. coli) K12, AIEC-HM605, and Salmonella typhimurium, short-chain fatty acid (SCFA) mixture (acetate, propionate, butyrate), and PBS control.

*, **, *** p < 0.05, p < 0.01, p < 0.001 compared to Control (PBS).

#, ## p < 0.05, p < 0.01 compared to LB broth.

### Pro-inflammatory Cytokines Induce the Expression of CEACAM1, CEACAM5, and CEACAM7 in a Time-Dependent Manner and Sustains the Expression of CEACAM3 and CEACAM6 Over Time in Intestinal Epithelial Cells

**Figure 5:**
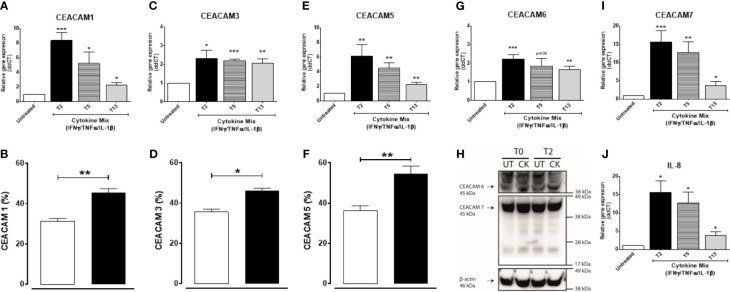
C2BBe1 cells cultured for 3 h with the pro-inflammatory cytokine cocktail containing recombinant human (rh) TNF-α (20 ng/ml), rh IFN-γ (10 ng/ml), rh IL-1β (10 ng/ml), and untreated. After the 3 h co-culture (T0), cells are washed followed by further culture in full culture media for 2 (T2), 5 (T5), and 13 (T13) h. Samples are collected at T0, T2, T5, and T13 time points for analysis on gene and protein expression of **(A, B)** CEACAM1, **(C, D)** CEACAM3, **(E, F)** CEACAM5, **(G, H)** CEACAM6, **(H, I)** CEACAM7, and **(J)** IL-8 as determined by RT q-PCR, flow cytometry (CEACAM1, -3, -5), WB (CEACAM6 and 7). For gene expression analysis, graphs are representative of three to five independent experiments with pooled triplicate samples and RT-qPCR assay performed in duplicates. β-actin was used as a housekeeping gene, and results are expressed as fold change. For protein analysis with flow cytometry and WB, graphs are representative of two independent experiments with pooled duplicates/triplicates. In the flow cytometry graphs **(B, D, F)** white colums represent untreated samples and black columns represent cytokine cocktail treated samples collected at T5. A Student t-test was used to analyze gene and protein data. *P < 0.05, **P < 0.01, ***P < 0.001 when compared to untreated cells. UT, untreated; CK, cytokine cocktail treatment.

We next examined whether a pro-inflammatory environment characterized by the pro-inflammatory cytokines IFNγ, TNFα, and IL-1β, all associated with IBD-pathophysiology, would alter the expression of CEACAMs. IECs are treated with a cytokine cocktail for 3 h, and expression of CEACAMs were assayed at T2, T5, and T13. Culture of IECs with cytokine cocktail results in a significant induction of CEACAM1, -5, -7 and IL-8 at T2, followed by a reduced gene expression over time (**Figures 5A, E, I, J**). Interestingly, the expression of CEACAM3 and CEACAM6 is sustained over time in IECs cultured with the cytokine cocktail (**Figures 5C, G**). To validate the gene expression results, protein levels of the five CEACAMs are evaluated by flow cytometry and western blotting. The protein levels of CEACAM1, -3, -5 are significantly increased at T5 compared to untreated cells (**Figures 5B, D, F**) and are reduced at T13. In contrast, protein levels of CEACAM6 and -7 are detected at the earlier time points T0 and T2 (**Figure 5H**) and reduced at T5.

### The JAK Inhibitor Tofacitinib Reduces the Expression of Cytokine-Induced CEACAMs in Intestinal Epithelial Cells

**Figure 6:**
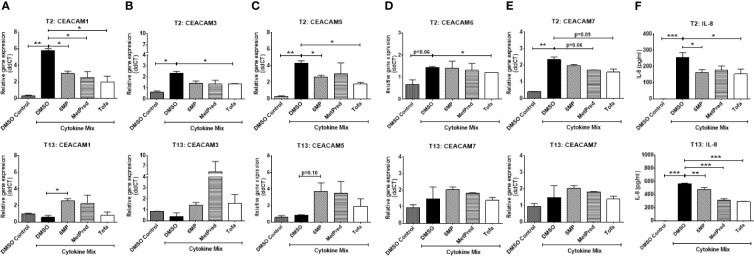
C2BBe1 cells were pretreated for 1 h with the IBD drugs—Tofacitinib (Tofa, 10 mM), Methylprednisolone (MetPred, 10 mM), Mercaptopurine (6-MP, 5 mM)—followed by culture for 3 h with the pro-inflammatory cytokine cocktail (Cytokine Mix), containing recombinant human (rh) TNF-α (20 ng/ml), rh IFN-γ (10 ng/ml), rh IL-1β (10 ng/ml), and untreated. After the 3 h co-culture, cells are washed in medium followed by further culture in full culture media containing respective IBD-drug for 2 (T2) and 13 (T13) h. Control wells (DMSO Control) are treated with 0.1% DMSO. Samples are collected at T2 and T13 time points for analysis on gene expression of **(A)** CEACAM1, **(B)** CEACAM3, **(C)** CEACAM5, **(D)** CEACAM6, **(E)** CEACAM7 as determined by RT q-PCR and **(F)** IL-8 determined by ELISA. Graphs are representative of two independent experiments with pooled triplicate samples and qRT-PCR assay performed in duplicates. β-actin was used as a housekeeping gene, and results are expressed as fold change. A Student t-test was used to analyze gene expression data. *P < 0.05, **P < 0.01, ***P < 0.001 when compared outlined treatment.

Next, we ask whether IBD drugs known to induce remission in the patients could affect the cytokine-induced expression of CEACAMs in IECs. IECs are pretreated with the IBD drugs Tofacitinib (Tofa), Methylprednisolone (MetPred), and Mercaptopurine (6-MP) for 1 h, followed by culture with the cytokine cocktail (IFNγ, TNFα, and IL-1β) for 3 h and collection of cells at T2 and T13 to assay the expression of CEACAMs and IL-8 secretion. At T2, Tofacitinib significantly reduces the expression of most cytokine-induced CEACAMs (CEACAM1, -3, -5, -6; **Figures 6A–D**), while 6-MP and MetPred significantly reduce the expression of cytokine-induced CEACAM1, -5 and CEACAM1, -6, respectively (**Figures 6A, C, D**). The three drugs significantly reduce IL-8 secretion (**Figure 6F**). At T13, no significant difference in the expression of cytokine-induced CEACAMs is observed by Tofacitinib, although IL-8 secretion is significantly reduced (**Figures 6A–F**). Interestingly, both 6-MP and MetPred induce the expression of CEACAM1, -3, -5 when compared to IECs treated to cytokine cocktail (**Figures 6A–C**), while both drugs reduce the secretion of IL-8 at T13 (**Figure 6F**).

## Discussion

This study explores the potential role of intestinal CEACAMs in early-onset and in chronic adult IBD with a particular focus on the impact of IBD-associated triggers on the expression of CEACAMs and correlation to inflammation and response to IBD drugs.

Our data identify, for the first time, the increased expression of CEACAM3, -5, -6 in colon biopsies of treatment-naïve pediatric patients with CD and in adult patients with UC. Culture of IECs with a pro-inflammatory cytokine cocktail and the CD-pathobiont AIEC results in a rapid induction of CEACAM1, -3, -6, -7, correlating to IL-8 expression; while the emulsifier p80 increased the expression of all CEACAMs correlated to increased cell death. In contrast, SCFA treatment significantly reduced CEACAM1, -5, -7 expression. Finally, cytokine-induced CEACAMs’ expression and IL-8 secretion were reduced in IECs treated with three IBD drugs. Collectively, these findings indicate that CEACAM3, -5, and -6 may play a role in IBD pathogenesis, especially in the early onset of colonic CD and in chronic UC.

It is intriguing that the different IBD triggers studied herein can similarly regulate the expression of CEACAMs, with most correlating to the degree of inflammation. CEACAMs have been identified as receptors for certain bacteria including CEACAM6 for AIEC (12); CEACAM1, -5, -6 for *S. typhimurium (* 19); and CEACAM1,-3,-6 for *Neisseria* spp. Binding of *Neisseria* spp. *via* the colony opacity-associated (Opa) outer membrane proteins results in an oxidative burst and degranulation due to the phosphorylation of immunoreceptor tyrosine-based activation motif (ITAM) by tyrosine kinase Syk activation in neutrophils and epithelial cells (30, 31). CEACAM3 can also serve as a receptor to DraE adhesins found on *E. coli*, with the potential to recruit CEACAM3 to bacteria bound to other receptors (32). Although AIEC infection of IECs results in a relatively low expression of CEACAM3, treatment with pro-inflammatory cytokines induces its gene and protein expression, which can contribute to the recognition of AIEC or other unknown pathobionts, leading to increased IL-8 secretion, recruitment of neutrophils, and potentiation of chronic inflammation. In contrast to AIEC and cytokine treatment, the p80 emulsifier provokes an early and weak induction of IL-8 in IECs, in line with previous studies (5, 11), followed by a late induction of CEACAM3 expression. Recent reports indicate that p80 can affect the intestinal barrier and mucus layer (33) and can increase the expression of several CEACAMs, including CEACAM3, -6 (this study), thereby allowing bacteria such as AIEC to penetrate and induce and sustain a heightened immune response. Overall, these observations indicate a potential role of trigger-induced CEACAM3 in the pathogenesis of IBD. Future studies should screen IBD-associated bacteria for their ability to bind to CEACAM3 and identify mechanisms regulated by bacteria–CEACAM3 interactions in intestinal inflammation.

Over two decades ago, the AIEC strain LF82 was identified and isolated from the mucosa of patients with CD, and today it is considered the most putative bacteria candidate in CD pathogenesis (13, 14). CEACAM6 was identified as a receptor to which AIEC can adhere to by using FimH adhesin, leading to its colonization of the gut mucosa and subsequent inflammation (12). CEACAM6 was upregulated in ileal epithelial cells of patients with CD and in IECs cultured with IFNγ or the AIEC-LF82 strain and to a lesser degree with TNFα (12, 14). In our study and similarly to Barnich et al., pro-inflammatory cytokines induce CEACAM6 expression in IECs, while AIEC-HM605 did not. The difference in the autocrine regulation of CEACAM6 by the two AIEC strains may be related to their bacterial properties, e.g., LF82 is an ileal isolate and dependent on type 1 pili (13, 14), while HM605 is a colonic isolate, expresses hemagglutinins, and is phylogenetically distant to LF82 (34, 35) or the colon cell line (C2BBe1) used in this study. In line with this, the pathoadaptative mutation capacity of FimH can result in a variety of distinct allelic *fimH* variants (12, 14), prompting us to speculate that AIEC-HM605 may express a low-infective FimH allelic variant, rendering a lower bacterial adhesion capacity to IECs when compared to LF82 strain and therefore resulting in a lower CEACAM6 induction. Another explanation for the low CEACAM6 expression induced by pro-inflammatory cytokines or AIEC-HM605 could be the degree of differentiation of the cells. Previous studies have shown an increased expression of CEACAM6 in polarized epithelial cells and long-term treatment with IFNγ (2–4 days) (12, 36). We confirm previous findings on CEACAM6 in the adult CD samples and expand them by reporting an increase in CEACAM6 expression in pediatric colon CD. Interestingly, other studies report an up to 10% abundance of AIEC in pediatric CD (13, 37, 38), further supporting a role for AIEC–CEACAM6 interaction for CD onset. In contrast to Barnich et al. (12), we observe a significant increase in CEACAM6 in colon biopsies from patients with UC, regardless of disease state, which can be provoked by other IBD triggers such as pro-inflammatory cytokines and emulsifiers. Although AIEC has been mostly associated to CD, recent reports indicate that AIEC can also be found in the UC mucosa. A recent systemic review and meta-analysis report a prevalence of AIEC in 12% of patients with UC compared to 29% in patients with CD and 5% in non-IBD controls, although with quite a varied range in all groups (38, 39). CEACAM6 expression was not increased in pediatric UC samples but in adult UC samples, indicating that AIEC may be a potential factor in sustaining chronic inflammation in UC. Overall, our data validate CEACAM6 as an underlying molecular player in colonic IBD, where an inflammatory and favorable environment created by, e.g., pro-inflammatory cytokines and emulsifiers can provide a constant anchor for increased pathobiont-CEACAM6 interactions on epithelial cells for onset and sustaining of IBD.

In contrast to two previous reports showing a reduction in CEACAM7 expression in IBD (19), we observed no major changes in CEACAM7 expression in colon biopsies of adult and pediatric IBD patients. Notably, a negative correlation to IL-8 in UC patients was observed, suggesting that inflammation might negatively affect the regulation of CEACAM7 expression in these patients. Furthermore, we showed that pro-inflammatory cytokines and AIEC co-culture induce a rapid CEACAM7 gene and protein expression followed by a reduction over time in IECs. These findings are in line with previous studies in colon cancer patients, where an initial upregulation of CEACAM7 was followed by a rapid decrease over time (25). This could also be reflective of a different transcriptional regulation for CEACAM7 *versus* other CEACAMs. As CEACAM7 levels in cancer patients correlate with the duration of their cancer diagnosis (25), future studies should investigate CEACAM7 alterations in long-term sufferers of UC, who are at a higher risk to develop colon cancer, as a potential diagnostic biomarker.

Previous reports indicate CEACAM5 expression is increased in adult UC biopsies and reduced in epithelial cells and colon biopsies of adult patients with CD (21, 24). In agreement with these findings, we note a significant increase in adult UC and a reduction in adult CD compared to UC biopsies and no changes compared to adult controls. Furthermore, we report an increase in CEACAM5 in pediatric CD samples and an induction by pro-inflammatory cytokines, p80, and AIEC in IECs. Previous studies have indicated that CEACAM5 can promote inflammation through the inhibition of the CD8^+^ suppressor T cells *via* gp180-CEACAM5 interaction, preventing the suppression of T helper (Th)1 cells (21), potentially indicating a role for CEACAM5 in the early onset of CD and in chronic UC.

CEACAM 1 is widely distributed in the body and particularly in IECs. Inhibition of CEACAM1 has been shown to reduce inflammation by inhibiting T cell responses in experimental models (19). Our study identified an early induction of CEACAM1 by pro-inflammatory cytokines, AIEC, and p80 and in pediatric CD biopsies, without major alterations in pediatric UC or adult IBD biopsies, indicating a potential role for CEACAM1 in the onset of CD. In colon cancer, a dynamic regulation of CEACAM1 appears dependent on the stage of the disease (40), which might potentially reflect the CEACAM profile seen in pediatric *versus* adult IBD. Although CEACAM1 was reported to be partially regulated by the commensal microbiota in mice, our data do not support this notion (20).

Co-culture of IECs with the intestinal pathogen *S. typhimurium* or a commensal *E. coli* strain has very little impact on CEACAM expression, even though *S. typhimurium* induced an inflammatory response and it can be recognized by CEACAM1, -5, -6, indicating there is no autocrine regulation of CEACAMs by *S. typhimurium*. A potential role of the microbiota regulating CEACAMs was reported in mice, where Gram-positive bacteria but not Gram-negative regulated the expression of CEACAM1 and CEACAM20 in the small intestine and epithelial cells, although both CEACAM1 and 20 are also highly expressed in the colon (20). Using germ-free or microbiota-depleted humanized transgenic mouse CEABAC10, expressing human CEACAM3, -5, -6, and -7, would provide an insight on the role of the commensal microbiota in the regulation of these CEACAMs’ expression and intestinal location.

When selected IBD drugs known to induce remission in IBD patients are evaluated for their regulation on cytokine-induced CEACAMs expression, it reveals that the JAK-inhibitor Tofacitinib efficiently inhibits cytokine-induced CEACAM expression in IECs. The JAK-STAT pathway plays a critical role in regulating the immune response, especially cytokines and T cell responses, and is highly associated to IBD pathology, and targeting this pathway using small molecules such as Tofacitinib have shown efficacy in patients with UC but not CD (7, 41). Previous studies have shown that IFNγ treatment of IECs regulates the expression of CEACAM1, -5, -6 (12, 36), supporting an interaction between CEACAMs and JAK/STAT pathway and our findings with Tofacitinib. Upacitinib, a JAK1 selective inhibitor, has proven efficacious in CD patients. A recent study indicates that Upacitinib treatment induces similar remission response in patients with ileal and colon CD; however, at the transcriptional level, the gene regulation by Upacitinib is more pronounced in the colon than in the ileum (42). Interestingly, 60 genes, including CEACAM3, are equally regulated by Upacitinib in both ileum and colon of these patients (42), potentially indicating that JAK inhibitors can regulate similar CEACAM mechanisms at different intestinal locations. Future studies should further dissect the regulation of CEACAM expression by the JAK-STAT pathway using clinically available JAK inhibitors to identify their role in IBD and colon cancer.

## Conclusions

In summary, we have shown that the expressions of CEACAMs are altered in colonic biopsies of both pediatric and adult patients with IBD and are upregulated by known IBD triggers. Collectively, our data indicate that an inflammatory environment caused by different IBD triggers can result in increased expression of CEACAMs, which can recognize pathobionts such as AIEC, thereby providing a favorable environment for their survival and perpetuation of inflammation. Overall, we provide new evidence on the regulation of CEACAM expression by IBD triggers and their contribution to IBD pathogenesis.

## Data Availability Statement

The raw data supporting the conclusions of this article will be made available by the authors, without undue reservation.

## Ethics Statement

The studies involving human participants were reviewed and approved by University College Cork Clinical Research Ethics Committee of Cork Teaching Hospitals; the Institutional Research Ethics Committee, Our Lady’s Children’s Hospital Crumlin. Written informed consent to participate in this study was provided by the adult paticipants and by the participants’ legal guardian/next of kin.

## Author Contributions

GS-G carried out experiments, analyzed data, and prepared and revised the manuscript. NH, VR, RS, MA, MK, SHi, RO’S, and AF carried out experiments and analyzed the data. PTW, SHu, FS, and KN recruited patients and coordinated the collection of human colon biopsies from adult and pediatric individuals. FS, KN, and SM secured funding. CO’D supervised and reviewed the research and manuscript. SM conceived, directed, and supervised the research and wrote the manuscript with valuable input from all authors. All authors contributed to the article and approved the submitted version.

## Funding

This work and the authors were supported by a Science Foundation Ireland (SFI) Research Centre awards SFI/12/RC/2273-P1 and SFI/12/RC/2273-P2 to APC Microbiome Ireland; and the National Children’s Research Centre Project award K-17-1 to PW and SHu. GS-G is a recipient of a Government of Ireland Postgraduate Scholarship (grant GOIPG/2019/4528). RS receive financial support from grant number SFI/15/RP/2828. The DOCHAS study is supported by the National Children’s Research Centre, Crumlin, Dublin.

## Conflict of Interest

FS - Co-founder/shareholder of Alimentary Health Ltd, Tucana Health Ltd and Atlantia Food Clinical Trials Ltd. Scientific advisor to Kaleido Biosciences.

The remaining authors declare that the research was conducted in the absence of any commercial or financial relationships that could be construed as a potential conflict of interest.

## Publisher’s Note

All claims expressed in this article are solely those of the authors and do not necessarily represent those of their affiliated organizations, or those of the publisher, the editors and the reviewers. Any product that may be evaluated in this article, or claim that may be made by its manufacturer, is not guaranteed or endorsed by the publisher.

## Supplementary Material

Supplementary Figure 1 **(A)** Representative side scatter plot *versus* live/dead staining. Numbers in the quadrant represent the live cells. **(B)** Representative live/dead *versus* anti-CEACAM1, anti-CEACAM3, anti-CEAM5 (as indicated) dot plots of epithelial cells (C2BBe1), after gating on live cells. Numbers in quadrants indicate percentage of respective quadrant population.
