# Supplementary figures and images for: Regulation of CEACAM Family Members by IBD-Associated Triggers in Intestinal Epithelial Cells, Their Correlation to Inflammation and Relevance to IBD Pathogenesis
Source: Front Immunol. 2021 Jul 29;12:655960. doi: 10.3389/fimmu.2021.655960 (PMC8358819; doi:10.3389/fimmu.2021.655960)

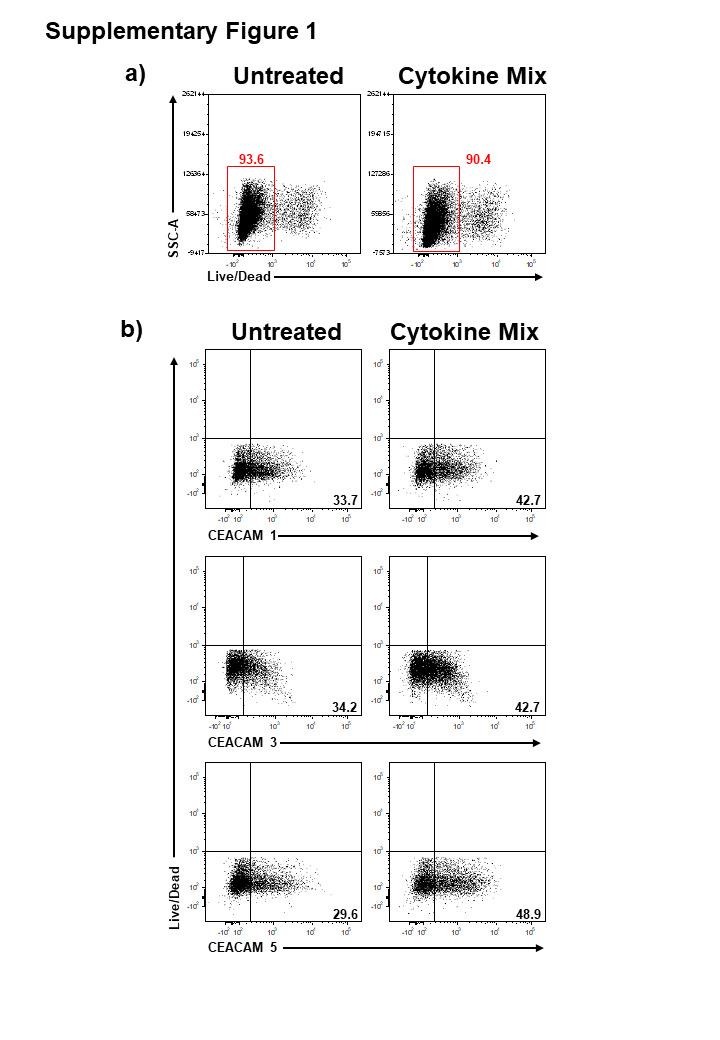

Supplement: Supplementary Figure 1 — (A) Representative side scatter plot versus live/dead staining. Numbers in the quadrant represent the live cells. (B) Representative live/dead versus anti-CEACAM1, anti-CEACAM3, anti-CEAM5 (as indicated) dot plots of epithelial cells (C2BBe1), after gating on live cells. Numbers in quadrants indicate percentage of respective quadrant population. [file Image_1.tif]
