# Supplementary material for: Regulation of CEACAM Family Members by IBD-Associated Triggers in Intestinal Epithelial Cells, Their Correlation to Inflammation and Relevance to IBD Pathogenesis
Source: Front Immunol. 2021 Jul 29;12:655960. doi: 10.3389/fimmu.2021.655960 (PMC8358819; doi:10.3389/fimmu.2021.655960)
Supplement: Supplementary file 2 [file DataSheet_1.docx]

**Supplementary Table 1. Clinical characteristics of Pediatric patients included in the study at the time of biopsy collection**

| Variables | | Control | CD | UC |
| --- | --- | --- | --- | --- |
| Mean Age | (years) | 6.7 ± 5.1 | 11.6 ± 2.2 | 12.4 ± 3.3 |
| Sex | Male | 5 (83.3%) | 7 (58.3%) | 6 (66.7%) |
|  | Female | 1 (16.1%) | 5 (41.7%) | 3 (33.3%) |
| Disease State | Mild | N/A | 2 (16.7%) | 4 (44.4%) |
|  | Moderate | N/A | 3 (25.0%) | 2 (22.2%) |
|  | Severe | N/A | 7 (58.3%) | 3 (33.3%) |
|  |  |  |  |  |

**Supplementary Table 2 - Clinical characteristics of Adults patients included in the study at the time of biopsy collection**

| Variable | | Control | CD Active | CD Inactive | UC Active | UC Inactive |
| --- | --- | --- | --- | --- | --- | --- |
| Mean Age | (years) | 45.6 ± 12.6 | 33.2 ± 10.8^a^ | 47.0 ± 15.0 | 50.8 ± 13.7 | 47.0 ± 15.0 |
| Sex | Male | 4 (28.6%) | 3 (21.4%)^b^ | 3 (30%) | 10 (52.6%) | 10 (52.6%) |
|  | Female | 10 (71.4%) | 9 (64.3%) | 7 (70%) | 9 (47.4%) | 9 (47.4%) |
| Medications | Aminosalicylates | 0 (0%) | 6 (50%) | 5 (50%) | 7 (36.8%) | 16 (84.2%) |
|  | Immunosuppressant | 0 (0%) | 3 (25%) | 3 (33.3%) | 2 (10.5%) | 2 (10.5%) |
|  | Steroids | 0 (0%) | 3 (16.7%) | 3 (30%) | 2 (10.5%) | 1 (5.3%) |
|  | Anti-TNFs | 0 (0%) | 1 (8.3%) | 2 (20%) | 1 (5.3%) | 0 (0%) |
|  | None | 0 (0%) | 1 (8.30%) | 1 (10%) | 3 (15.8%) | 2 (10.5%) |
|  | Other | 4 (33.3%) | 5 (41.7%) | 2 (20%) | 4 (21.1%) | 9 (47.4%) |
| Median Disease Duration (years, range) |  | N/A | 26 (11-42) | 32.5 (21-66) | 37 (10-62) | 30 (5-49) |

^a^ UC active vs CD active; p < 0.01

^b^ UC active vs CD active, males; p < 0.05

**Supplementary Table 3 – Sequences of primers and probes used in this study**

|  | **Left Primer** | **Right Primer** | **Probe** |
| --- | --- | --- | --- |
| CEACAM1 | 5’ – GATTTGCCATAGCCTTGAGGT – 3’ | 5’ – GGCATTACTGCCTTTACTTTCTCT- 3’ | 72 |
| CEACAM3 | 5’ - ACTGGACAGTTCCATGTATACC - 3’ | 5’ - TTGTCCTCCTTGGGGTTG - 3’ | 67 |
| CEACAM5 | 5’ – TTCCATAGTCAAGAGCATCACAG – 3’ | 5’ – CCAACCAGCACTCCAATCAT – 3’ | 57 |
| CEACAM6 | 5’ – GCATCACGATTGGAGTGCT – 3’ | 5’ – AGGGTCTGGTCCAATCTGC – 3’ | 21 |
| CEACAM7 | 5’ – TGGTGGGTAAACAATCAGAGC – 3’ | 5’ – GGGTCCTGTTGTCAGTGGAG – 3’ | 64 |
| β- Actin | 5’ – ATTGGCAATGAGCGGTTC – 3’ | 5’ – CGTGGATGCCACAGGACT – 3’ | 11 |
| IL-8 | 5’ - ATGGTTCCTTCCGGTGGT - 3’ | 5’ - GAGCACTCCATAAGGCACAAA - 3’ | 72 |
